# Dissection of physiological, transcriptional, and metabolic traits in two tall fescue genotypes with contrasting drought tolerance

**DOI:** 10.1002/pei3.10066

**Published:** 2021-11-22

**Authors:** Yun Kang, Shyamal Talukder, Zewei An, Ivone Torres‐Jerez, Nick Krom, David Huhman, Michael Udvardi, Malay C. Saha

**Affiliations:** ^1^ Noble Research Institute, LLC Ardmore Oklahoma USA; ^2^ Institue for Agricultural Biosciences Oklahoma State University Ardmore Oklahoma USA; ^3^ Texas A&M AgriLife Research Center Beaumont Texas USA; ^4^ State Center for Rubber Breeding and Rubber Research Institute Danzhou Hainan China; ^5^ The University of Oklahoma Health Sciences Center Oklahoma City Oklahoma USA; ^6^ Queensland Alliance for Agriculture and Food Innovation The University of Queensland Brisbane Queensland Australia

**Keywords:** drought, endophyte, metabolomics, proline, tall fescue, transcriptome

## Abstract

Tall fescue (*Festuca arundinacea*) is an important cool‐season perennial forage grass that forms mutualistic symbioses with fungal endophytes. Physiological, biochemical and transcriptional comparisons were made between two tall fescue genotypes with contrasting drought tolerance (tolerant, T400, and sensitive, S279), either with or without endophyte (*Epichloë coenophiala*). Drought stress was applied by withholding watering until plants reached mild, moderate and severe stresses. Physiological characterization showed that T400 had narrower, thicker leaves, and lower leaf conductance under well‐watered conditions, compared to S279. After severe drought and recovery, endophytic T400 had greater shoot and root biomass than other plant types. Under drought, leaf osmotic pressure increased much more in T400 than S279, consistent with accumulation of metabolites/osmolytes, especially proline. Gene Ontology enrichment analysis indicated that T400 had more active organic acid metabolism than S279 under drought, and implicated the role of endophyte in stimulating protein metabolism in both genotypes. Overall T400 and S279 responded to endophyte differently in aspects of physiology, gene transcription and metabolites, indicating plant genotype‐specific reactions to endophyte infection.

## 1 INTRODUCTION

Tall fescue (*Festuca arundinacea*) is a cool season perennial grass. It is the most widely planted forage crop in the United States and covers almost 14 million ha (Sleper & West, 1996). Genetically it is an allohexaploid (2*n* = 6× = 42) cross‐pollinated species. Tall fescue evolved under a Medditerranean climate of hot, dry summers and cool, wet winters and, thus, performs well in the transitional zone of the United States, which includes a combination of cool/humid, cool/arid, warm/humid and warm/arid geographic zones. Tall fescue generally has better drought tolerance and/or avoidance mechanisms than other cool‐season perennial grasses such as ryegrass (*Lolium perenne* L.) and Kentucky bluegrass (*Poa pratensis* L.; Huang & Gao, 2000; Sheffer et al., 1987).

The persistence and performance of tall fescue is enhanced by symbiotic association with the fungal endophyte *Epichloë coenophiala* under various stress conditions, including drought (Bouton et al., 1993; Malinowski & Belesky, 2020; Pedersen et al., 1990). Endophytes enhance tall fescue tillering, root growth, aboveground biomass production, ability to absorb mineral phosphate from soil, osmotic adjustment, nitrogen utilization, and anti‐nematode activity (Assuero et al., 2002; Dinkins et al., 2019; Elmi et al., 2000; Panaccione et al., 2006). Thus, endophyte‐infected tall fescue has great potential as a forage crop in regions affected by episodic drought, amongst other environmental challenges.

Drought is the most important environmental factor limiting agriculture (Farooq et al., 2012). Plant responses to drought stress are complex and vary over space and time. Lack of water is perceived, in part, by membrane sensors in the root, which trigger systemic signaling pathways that affect gene expression throughout the plant. Plants have evolved diverse strategies to survive periods of drought, including developmental escape and avoidance, and biochemical tolerance (Fang & Xiong, 2015; Hirayama & Shinozaki, 2010; Meena & Kaur, 2019).

Drought resilience involves multiple traits, each typically controlled by multiple genes, which presents a major challenge for researchers and plant breeders interested in the underlying mechanisms and harnessing them to increase crop drought tolerance. Previous studies have identified common transcriptional responses to drought in various species, including induction of genes involved in transcriptional regulation, photosynthesis, hormone especially abscisic acid (ABA) metabolism, antioxidant biosynthesis, and metabolism of carbohydrate, amino acids, and fatty acids (Benny et al., 2019; Egea et al., 2018; Wang et al., 2017). Changes in both primary and secondary metabolites are associated with drought responses. Previous studies also point to important roles of osmolytes (e.g., trehalose, fructan and proline) as osmoprotectants under drought stress, among which, the importance of proline has been confirmed by various genetic studies (reviewed in Meena & Kaur, 2019).

Despite the importance of tall fescue as a primary forage species and of drought as a key limitation on forage production, the molecular and genetic mechanisms of drought tolerance in tall fescue remain largely unknown. Previous studies have explored physiological, biochemical and root developmental aspects of drought responses in tall fescue (Chen et al., 2018; Ebrahimiyan, , Majidi, Mirlohi, et al., 2013; Pirnajmedin et al., 2015; Saha et al., 2015; Sarmast et al., 2015; Sun et al., 2013) and the influence of endophytes on stress tolerance (Nagabhyru, Dinkins, Wood, et al., 2013). Only a few studies identified over/under‐expressed transcripts responsive to drought stress (Dinkins et al., 2019; Talukder et al., 2015). A systems study on the drought tolerance mechanism and the role of endophyte in tall fescue drought responses is still lacking.

Here, we investigated the physiological, biochemical, and transcriptional responses to drought stress of two tall fescue genotypes contrasting in drought tolerance, which were selected based on field and preliminary greenhouse experiments. We also determined the impact of symbiosis with the endophyte, *E. coenophiala* on drought responses in the two plant genotypes. We aimed to understand the systems mechanisms underlying the variance in tall fescue drought tolerance and the role of endophyte in this process.

## 2 MATERIALS AND METHODS

### 2.1 Plant material and growth conditions

The tall fescue genotypes T400 and S279 were selected from the tall fescue cultivar Texoma MaxQ II. Texoma MaxQ II is a tall fescue cultivar developed by the Samuel Roberts Noble Foundation in cooperation with AgResearch Ltd for its improved persistence and yield, and superior adaptation to the south‐central USA (Hopkins et al., 2011). In an earlier study exploring the genetic diversity related to drought tolerance of this cultivar, 1000 genotypes (plants) of Texoma MaxQ II were screened for drought tolerance in a greenhouse experiment using relative water content (RWC) and leaf osmotic potential (OP) as selection criteria. After initial screening, 25 selected most drought tolerant and susceptible genotypes were further evaluated in Ardmore, Oklahoma field for 2 years. These genotypes were also screened with PEG8000 in a greenhouse. Based on RWC and OP data obtained across all experiments, T400 and S279 were selected for its tolerance “T” and susceptibility “S” to drought stress, respectively, and used in the current study.

For T400 and S279, as well as all plants of Texoma MaxQ II, an asymptomatic novel fungal endophyte (*E. coenophiala*), AR584, lives in the intercellular space within the pseudostems (leaf sheath whorls). The endophyte makes a stable symbiosis with the plant and transmitted through seed. To generate endophyte‐free plants, the inherent AR584 endophyte in those genotypes was removed using a hydroponic system as described in Nagabhyru, Dinkins, Wood, et al. (2013). Both the developed clonal pairs were confirmed for endophyte status using immunoblot (An et al., 1993) and polymerase chain reaction assays (Takach et al., 2012).

Tall fescue plants were propagated by tillers and planted in tall plastic cones (35 × 7 cm, D60L, Stuewe and Sons., Inc.; https://www.stuewe.com). The soil was a mixture of metromix 360 and common sand (v/v = 2/1). At planting, two tillers were planted in each pot. The soil water content was monitored with EC‐5 soil sensors (https://www.metergroup.com/). For uniformity, the top edge of the soil sensor was 20 cm to the soil upper surface in each pot.

### 2.2 Drought treatment and plant sampling

Three weeks after being planted into the soil, ¾ of the plants were subjected to water withholding (drought‐stressed) and ¼ plants remained well‐watered (control). Drought‐stressed plants were harvested when the soil volumetric water content (VWC) reached 10% (mild‐stressed DrtA), 5% (moderately‐stressed, DrtB), and 1% (severely‐stressed, DrtC), respectively. The VWC of well‐watered plants (Ctl) were maintained at ~30%. To minimize variance, all samples were harvested between 1 pm and 2 pm each day. At harvest, the shoots and roots were collected separately and then frozen in liquid nitrogen immediately. The tissues were stored at −80°C until being ground in liquid nitrogen for RNA purification (RNAseq) and metabolite analysis gas chromatography–mass spectrometry (GC‐MS). For tissue collection to be used in quantification of the leaf OP, shoot/root dry weight and other physiological parameters, a separate drought experiment was performed.

### 2.3 Drought and re‐watering experiment

In a separate experiment from above, two tillers of each of the four plant types were planted in a three‐gallon plastic pot, two centimeters away from the edges avoiding the center of the pot, in random orders. A total of four pots and eight tillers of each plant type were used. When the soil VWC decreased to less than 1% and all the leaves lost chlorophyll and dried out, each pot was re‐watered and the plants were allowed to re‐grow for 20 days. At the end of re‐growth, shoots and roots were harvested separately and dried completely in a 55°C oven for dry weight quantification.

### 2.4 Leaf size and specific leaf weight

The size of the youngest fully‐expanded leaf of a tall fescue plant was measured with a Li‐3000A portable area meter (Li‐Cor; https://www.licor.com/). After area measurement, the leaf was completely dried in a 55°C oven and then the dry weight was quantified using a lab balance. The leaf specific weight was calculated by dividing the leaf area by the dry weight.

### 2.5 Guard‐cell density

The youngest fully‐expanded leaf of a tall fescue plant at harvest was collected and nail polish imprints were made of the middle session of the leaf, avoiding the edges. The imprints were subsequently observed and photographed under a microscope (Nikon TE300) at 100×. Stomata density was counted from photos.

### 2.6 Leaf conductance

Leaf conductance was measured with the SC‐1 Leaf Porometer (METER Group, Inc.; https://www.metergroup.com/) on the youngest fully‐expanded leaf. Each leaf was measured twice at the middle session and the average value was used.

### 2.7 In‐vivo leaf chlorophyll measurement

In‐vivo leaf chlorophyll content was measured with a Chlorophyll Meter SPAD‐502plus (Spectrum Technologies; http://www.specmeters.com/) on the youngest fully‐expanded leaf. Each leaflet was measured two times at the middle session and the average reading was used. The leaf edges were avoided at all measurements.

### 2.8 Leaf OP

The middle section (1 cm) of the youngest fully‐expanded leaf was sampled and then fully hydrated in sterile and de‐ionized water in a 2 ml Eppendorf tube for 48 h at 4°C. Next, the fully‐hydrated leaves were tap‐dried on a filter paper to remove surface water, and then stored at −80°C for over 24 h in a 0.65 ml Eppendorf tube. At the end of storage, a hole was punched at the bottom of the 0.65 ml Eppendorf tube and then it was placed inside a 1.5 ml Eppendorf tube, being centrifuged at 16,000 g for 10 min at 4°C to collect the leaf sap. The molal concentration of the leaf sap was measured at room temperature with a Wescor EliTechGroup Vapro 5600 Vapor Pressure Osmometer. Osmotic potential was calculated using the formula “OP = *iCRT*”, where *i* = ionization constant, *C* = Molal concentration (mole/kg), *R* = pressure constant (0.0831 liter bar/mole °K), *T* = temperature °K (273+°C).

### 2.9 Transcriptome analysis with RNAseq

Total RNA was isolated with the Spectrum™ Plant Total RNA Kit (Sigma) and then treated with TURBO DNA‐free™ Kit (Invitrogen) to remove DNA molecules. RNeasy MinElute Cleanup Kit (Qiagen) was used to further clean the DNase‐treated RNA samples. The quality of RNA samples was monitored by bio‐analyzer analysis using Agilent RNA 6000 Nano Kit (Agilent).

RNA samples were quantified using Qubit^®^ RNA BR (Broad‐Range) Assay Kit (Life Technologies). RNA‐seq libraries were prepared using TruSeq Stranded mRNA Sample Prep kits (Illumina). Individual libraries were uniquely indexed using TruSeq RNA Single Indexes (Illumina), and pooled in equimolar ratio. The pooled libraries were sequenced on a Hiseq4000 system (Illumina). The raw RNA‐seq data have been deposited to the NCBI Sequence Read Archive with reference number PRJNA746971.

All sequences were first quality trimmed using a custom Perl script which removed low quality bases (quality score < 30) from the ends of reads until two consecutive high‐quality bases were found. Reads less than 30 bp long after trimming were discarded, along with their mate pair. Each sample was then de novo assembled with Trinity version 2.2.0 (https://github.com/trinityrnaseq/trinityrnaseq). These independent assemblies were then merged by selecting sample CTL400P‐SH1 as a starting set, aligning the next (in alphabetical order) assembly with it using BLASTN, and adding the transcripts for which no homologs were found to the starting set. This process was repeated for every other sample, adding the unique transcripts from every other assembly to the starting set. Each sample's reads were then aligned to the merged transcript set with HISAT2 version 2.0.5 (http://ccb.jhu.edu/software/hisat2/index.shtml). The aligned reads were assembled and quantified using Stringtie version 1.2.4 (http://www.ccb.jhu.edu/software/stringtie/). For the purpose of obtaining functional annotations, the merged transcript set was aligned with the reference proteomes of Arabidopsis (TAIR 10), *Medicago truncatula* (IMGAG v4.0), and rice (MSU version 7). The annotation of each gene was assigned if being confirmed in at least two out of three plant species. Finally, all transcripts were filtered by at least 500 bp long, FPKM (Fragments Per Kilobase of exon per Million reads) >1 in at least one sample, and majority of non‐zero FPKM > 1. The normalized and filtered FPKM values were used in further analyses.

### 2.10 Metabolite analysis with GC‐MS

Metabolite analysis of polar and non‐polar metabolites were conducted following the procedure in Kang et al. (2011). Data analysis was performed using software MS‐DIAL (http://prime.psc.riken.jp/Metabolomics_Software/MS‐DIAL/).

### 2.11 Proline biochemical assay

Proline content was analyzed with a biochemical assay following Bates et al (Bates et al., 1973) and Hamid et al (Hamid et al., 2003). Proline concentration was determined using a standard curve generated using L‐proline.

### 2.12 Statistical analysis

For phenotypic and leaf osmotic pressure data, significant analysis was performed in R with package “agricolae”. Two‐way ANOVA (aov) was performed first and then Duncan's New Multiple Range Test was conducted for p value calculations. For GC‐MS and RNAseq data, significant analysis was performed by calculating *p* values with student's *t* test (two tails assuming equal variance) in excel. False discovery rate (FDR) adjusted *p* values (*p*_adj_) were calculated in R using function “fdr”.

### 2.13 Bioinformatic analysis

For having the best annotations, all bioinformatic analyses were performed using the closest *Arabidopsis thaliana* orthologs of corresponding tall fescue transcripts. List of drought regulatory gene illustration was performed using DiVenn 2.0 (https://divenn.tch.harvard.edu/; Sun et al., 2019). GO Enrichment analysis was performed in AgriGO v2 (http://systemsbiology.cau.edu.cn/agriGOv2/; Tian et al., 2017).

## 3 RESULTS

### 3.1 Physiological characterization of drought adaptation traits

**FIGURE 1:**
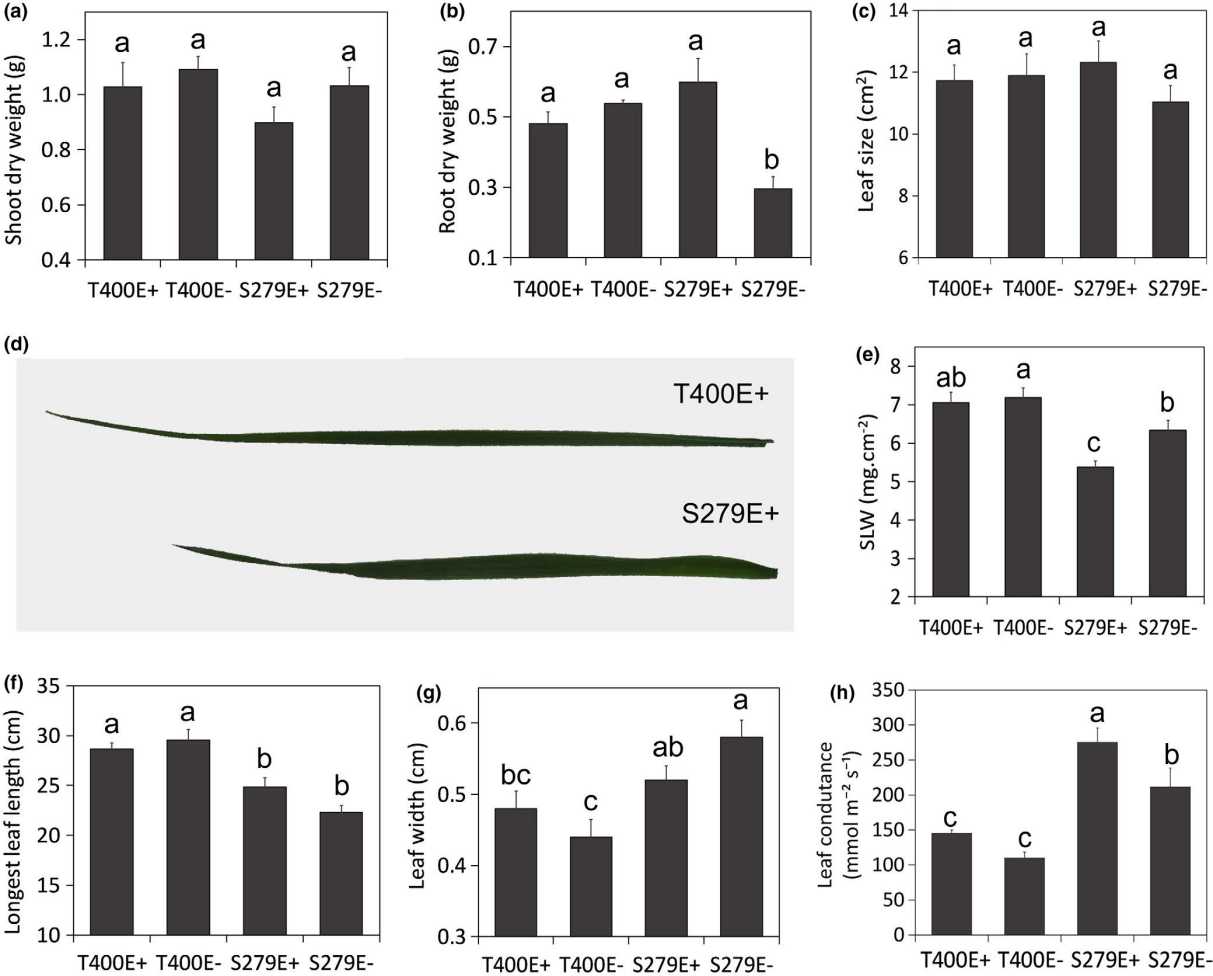
Biomass and leaf traits of well‐watered T400 and S279 plants. (a) Shoot dry weight, (b) root dry weight, (c) leaf size, (d) leaves of T400E+ and S279E+, (e) specific leaf weight (SLW), (f) leaf length, (g) leaf width, and (h) leaf conductance. Data were collected on four‐week‐old plants after one cut back. Different letters indicate significant difference at *p* < .05 (Duncan's test), *n* = 5, error bars are standard errors

Before performing drought stress experiments, we compared the shoot, root, and leaf phenotypes of the drought‐tolerant tall fescue genotype, T400, and the drought‐sensitive genotype, S279 under well‐watered conditions, with (E+) or without endophyte (E−). Shoot dry weights of the four plant‐endophyte combinations (T400E+, T400E−, S279E+, S279E−) were similar (Figure 1a). Interestingly, S279E+ invested significantly more in root growth than S279E− (Figure 1b; Figure S1). On the other hand, no significant difference was observed between T400E+ and T400E− in either shoot or root biomass (Figure 1a,b).

Leaf size and thickness of well‐watered plants were then compared. T400 had relatively long, narrow, and thick leaves, whereas S279 leaves were shorter, wider, and thinner (Figure 1c–g). The area of each leaf was similar among all plant‐endophyte combinations (Figure 1c). The difference between E+ and E− was not significant. Stomatal density on the abaxial side of leaves was similar among different plant types (Figure S2), while leaf conductance was significantly higher in S279 compared to T400 (Figure 1h).

**FIGURE 2:**
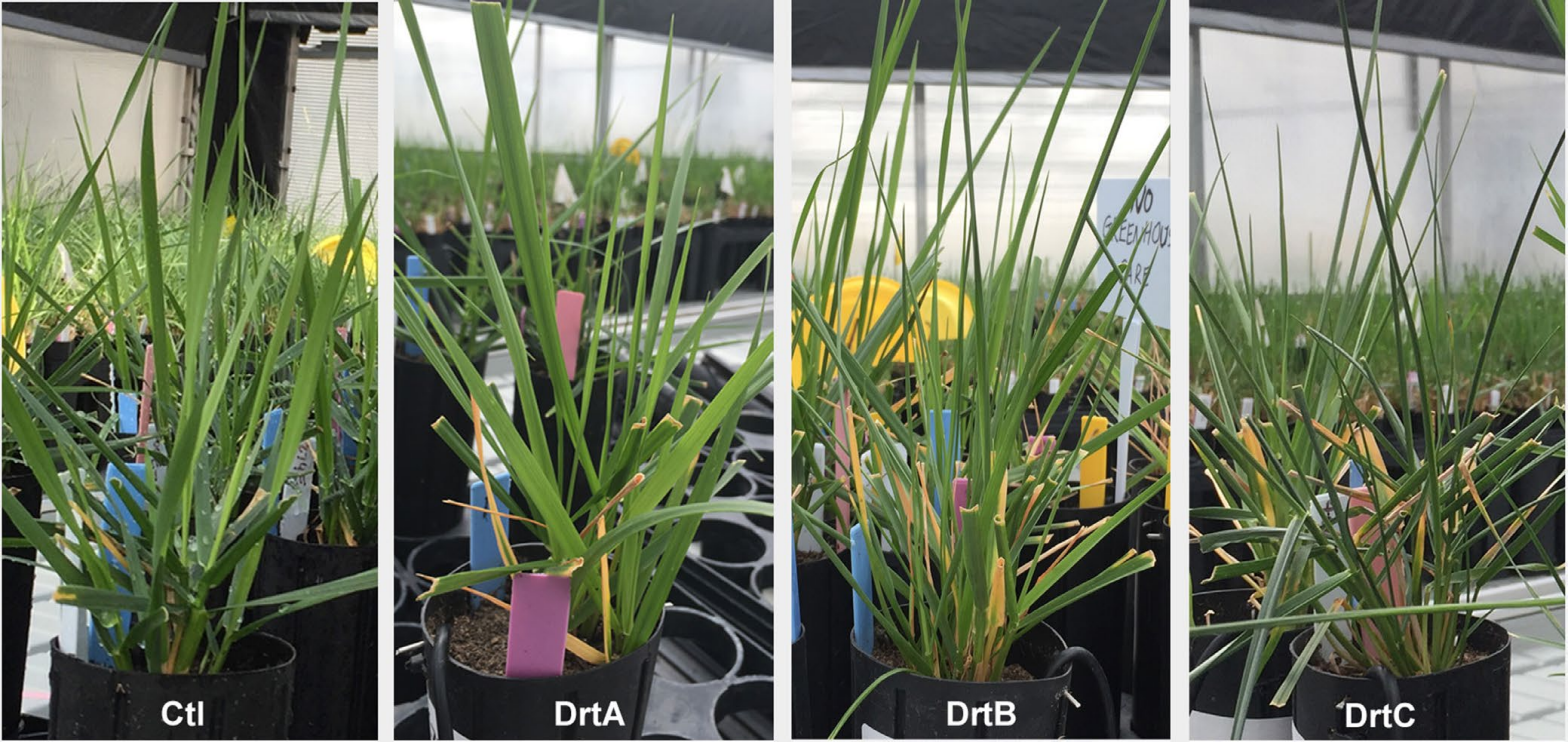
Well‐watered (Ctl), mild‐stressed (DrtA, soil VWC~10%), moderately‐stressed (DrtB, soil VWC~5%), and severely‐stressed (DrtC, soil VWC~1%) tall fescure plants (S279E+) at harvest. VWC, volumetric water content

**FIGURE 3:**
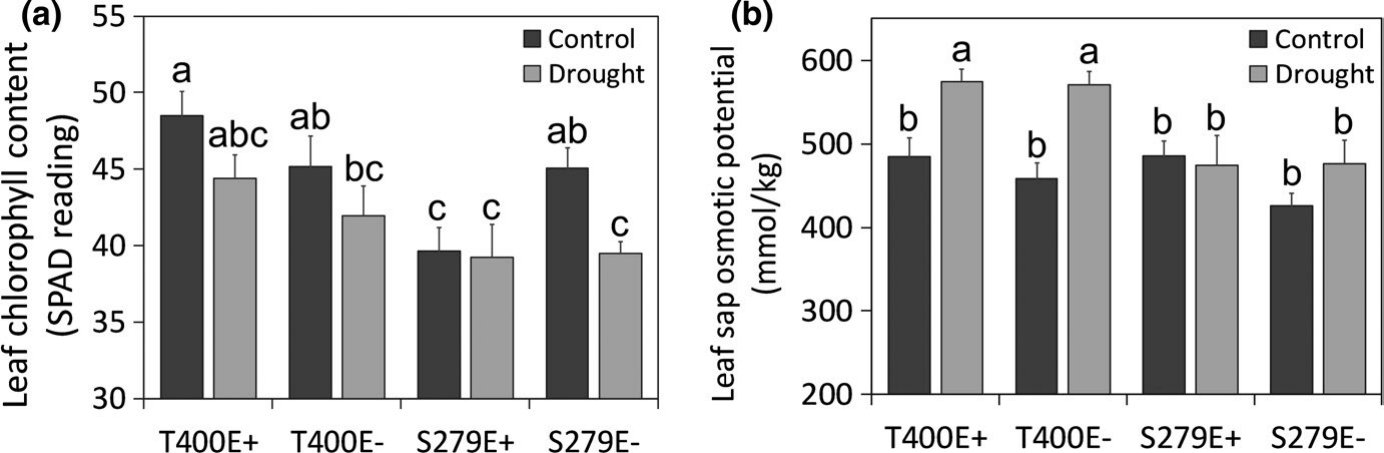
Leaf chlorophyll content (a) and leaf sap osmotic potential (b) of well‐watered and severely drought‐stressed tall fescue plants (soil VWC~1%). Different letters indicate significant difference at *p* < .05 (Duncan's test), *n* = 5, error bars are standard errors. VWC, volumetric water content

Drought stress was applied by withholding water. In a preliminary experiment, when soil VWC reached 10% (mild stress, DrtA), leaf gaseous water conductance decreased by 53% in S279E+ plants (274.1 ± 39.8 to 113.5 ± 17.8 mmol m^−^² s^−^¹, *n* = 4), although they appeared visibly similar to the well‐watered controls (Figure 2, DrtA). Leaf rolling was first evident at soil VWC of 5% (moderate stress, DrtB) and reached an extreme at 1% soil VWC (severe stress, DrtC; Figure 2). Under well‐watered conditions, T400E+ had the highest leaf chlorophyll content with 48.5 SPAD units, which was 18.2% higher than that of S279E+ with the lowest chlorophyll content. In addition, endophyte infection significantly reduced leaf chlorophyll content by 12% in S279, but did not cause significant changes in T400 (Figure 3a). Under severe drought stress, leaf chlorophyll content significantly decreased in S279E− but not in other plant types (Figure 3a). T400 and S279 had similar leaf OP at well‐watered conditions (Figure 3b). Under severe drought stress, the leaf OP of T400 was 19% (E+) to 24% (E−) higher than that of well‐watered controls, whereas no significant difference was observed in S279E+/− between drought and well‐watered conditions (Figure 3b). After severe drought stress, all plant types had similar shoot biomass, while S279E‐ had smaller root biomass than S279E+, which was similar to T400E+/− (Figure S3).

**FIGURE 4:**
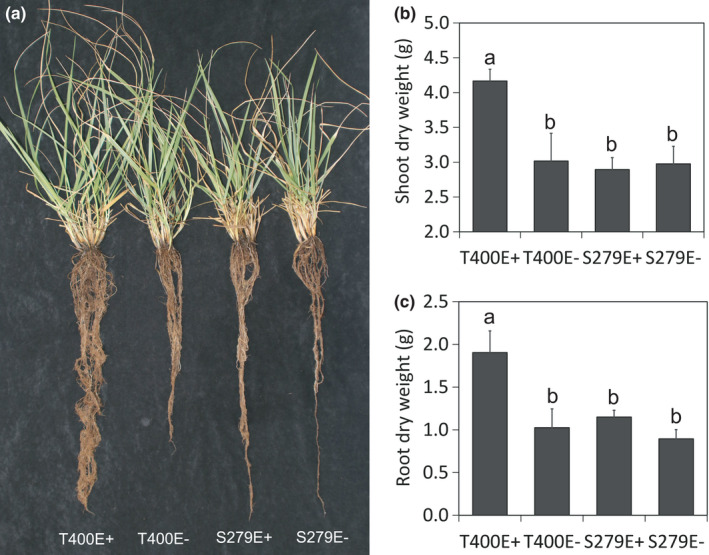
Tall fescue plants (a), shoot (b) and root (c) dry weight after severe drought stress (soil VWC < 1%) and recovering. Different letters indicate significant difference at *p* < .05 (Duncan's test), *n* = 5, error bars are standard errors. Image of plants in a pot is shown in (a). VWC, volumetric water content

In a separate experiment, the four tall fescue plant types were planted together in three‐gallon pots and the shoot/root biomass was measured after severe drought stress (<1% soil VWC) and recovery (Figure 4). After 20 days of recovery and re‐growth, T400E+ had much larger shoot and root biomass compared to other plant types, especially root biomass, which was nearly twice that of other plant types. No significant difference was observed between S279E+ and S279E−, either in shoot or root (Figure 4b,c).

### 3.2 Transcriptomic and metabolomic analyses of well‐watered and drought‐stressed tall fescue plants

**FIGURE 5:**
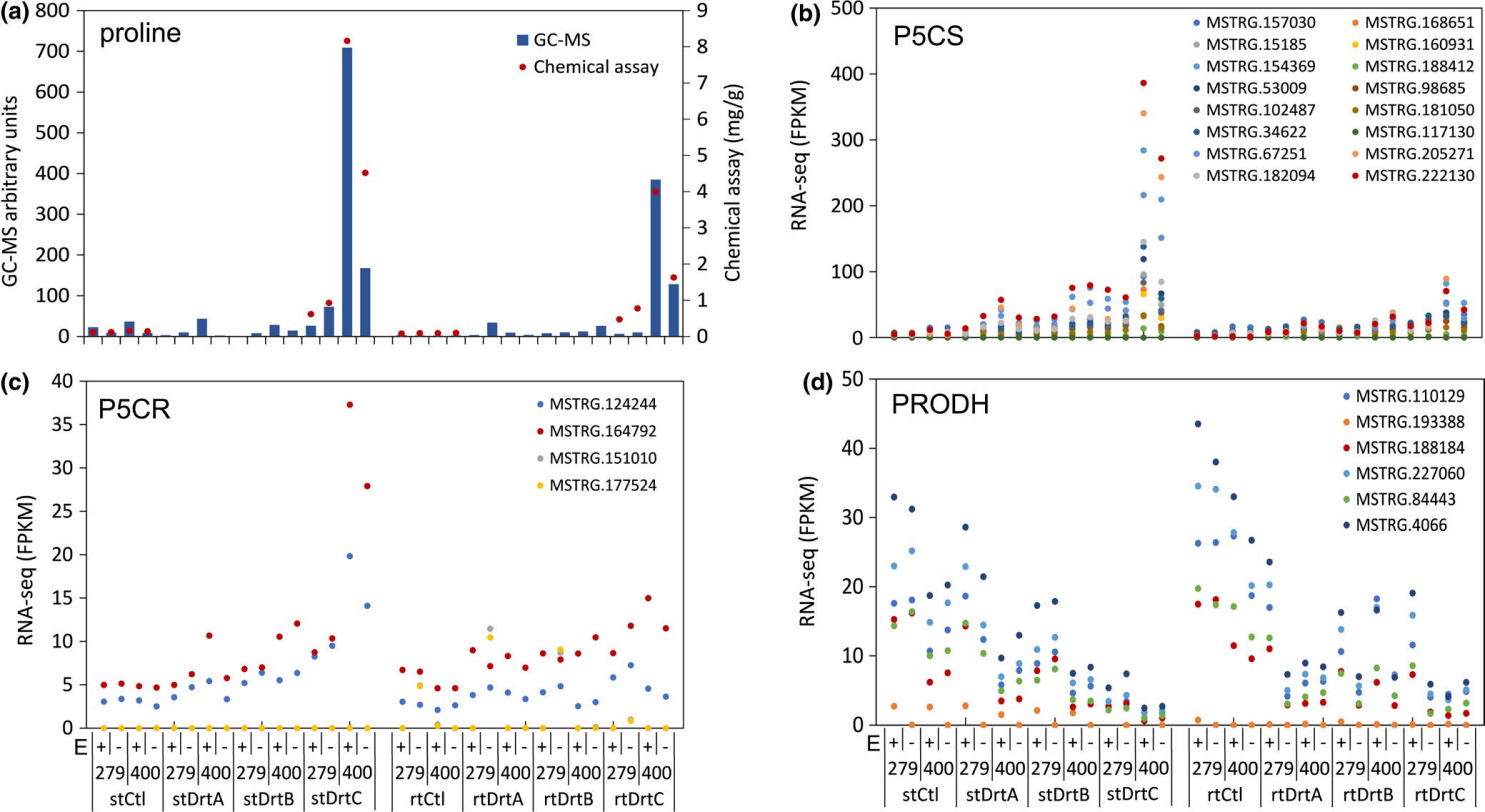
Accumulation of proline and transcript (FPKM) changes of major proline biosynthesis and degradation genes under drought stress. Average values of three replicates are shown. Nucleotide sequence of each transcript and annotation details (rice, Arabidopsis, and *M. truncatula*) are in Table S1. FPKM, Fragments Per Kilobase of exon per Million reads. E, endophyte; P5CS, delta‐1‐Pyrroline‐5‐carboxylate synthetase; P5CR, delta1‐Pyrroline‐5‐carboxylate synthase; PRODH, proline dehydrogenase; rt, root; st, shoot.

To gain insight into possible molecular and biochemical mechanisms underlying the contrasting physiological and developmental responses to drought stress between T400 and S279, and between E+ and E−, we performed RNAseq and GC‐MS analyses to examine transcriptomic and metabolomic changes, respectively. Under severe drought stress, a larger number of major polar metabolites accumulated in T400 shoots (17 metabolites) compared to S279 (eight metabolites), especially organic acids (Table 1). In the root, metabolite accumulation and depletion patterns were similar for S279E− and T400E+/−, while S279E+ roots appeared to be unique with the majority of metabolites decreasing in abundance compared to the control (Table 1). Among all major polar metabolites, proline and trehalose exhibited the greatest increase in relative abundance in response to drought in both roots and shoots of T400E+/− and S279E+/−. Because proline accumulated to much higher levels in T400 than S279 under severe drought, we further quantified levels of proline and analyzed transcripts of genes involved in proline biosynthesis and degradation (RNAseq results), under all stress conditions. Using a quantitative biochemical assay, proline levels were found to increase under drought stress in both shoots and roots, with the highest levels observed in severely‐stressed plants (DrtC; Figure 5). Severely‐stressed shoots of tolerant plants with endophyte, T400E+, had the highest proline levels (8.16 mg/g dry weight) among all samples. Transcript levels of the major proline biosynthetic enzyme, delta‐1‐Pyrroline‐5‐carboxylate synthetase (P5CS), but not delta1‐Pyrroline‐5‐carboxylate reductase (P5CR), mirrored the levels of proline, in both shoots and roots. In contrast, transcripts of proline dehydrogenase (PRODH), which mediates proline degradation, decreased with drought intensity, with the lowest levels under severe drought stress (Figure 5; Table S1).

**TABLE 1:**
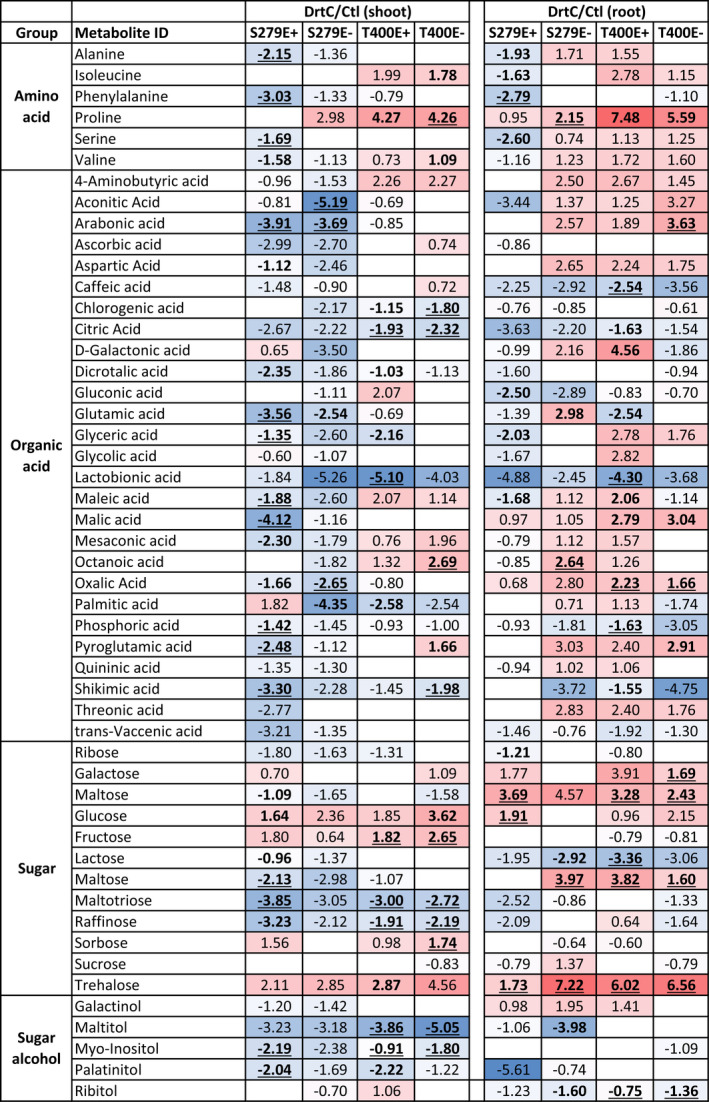
Major polar metabolite accumulation in severely drought‐stressed (DrtC) compared to well‐watered plants (Ctl). Log2 fold changes (FC) of metabolites are shown, with up‐ and down‐regulated metabolites colored in red and blue, respectively. FCs less than 1.5 (−0.85 < log_2_ratio < 0.85) are not shown. All FCs having *p* values less than .1 but equal to or larger than .05 are in bold only and FCs with *p* < .05 are in bold and underlined, *n* = 3

**FIGURE 6:**
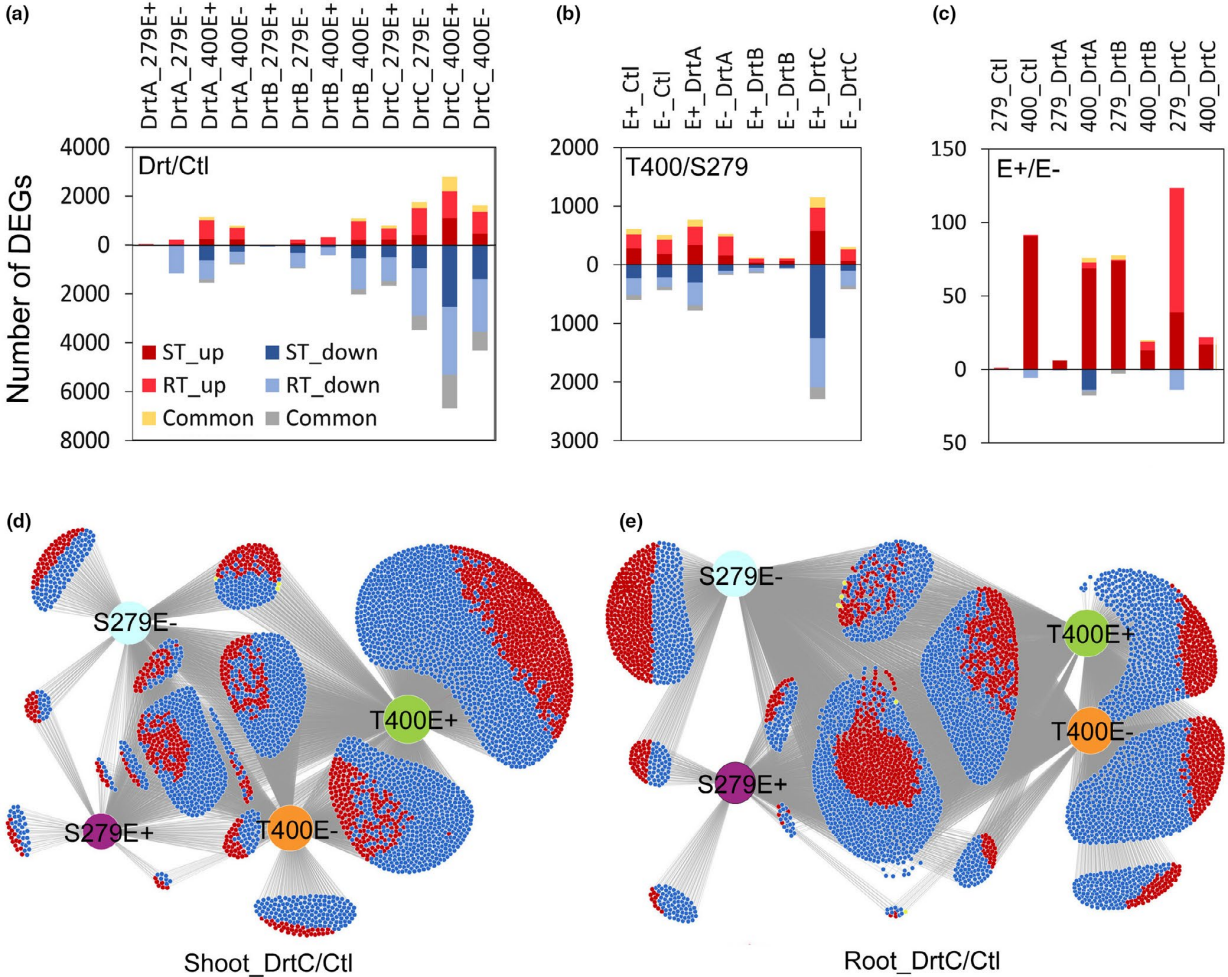
Numbers of differentially expressed genes (DEGs; FC > 2, *p*_adj_ < .05) that were regulated by drought stress (a), between T400 and S279 (b), and between E+ and E− (c). Severe drought stress (DrtC) regulated genes in shoots (d) and roots (e) are illustrated by a web‐based illustration tool Divenn (https://divenn.tch.harvard.edu/; Sun et al., 2019). Red denotes up‐regulated genes; blue denotes down‐regulated genes, and yellow denotes up‐ or down‐regulated genes

RNA‐seq analysis was carried out for plants with or without endophyte exposed to different levels of drought stress, to identify genes and associated biological processes affected by drought. Under drought stress, there were generally more down‐regulated than up‐regulated genes, and more differentially expressed genes (DEGs) in the roots than shoots (Figure 6a,d,e). Severely drought‐stressed T400E+ plants had the largest number of DEGs among all treatments, in both shoots and roots (Figure 6a). Comparing T400 and S279 (Figure 6b), severely drought‐stressed T400E+ also had the most number of DEGs compared with S279E+, with up to 1273 down‐regulated DEGs in the shoot (Figure 6d, e). The difference in transcript regulation between T400 and S279 was minimal under moderate stress (DrtB; Figure 6b). Numbers of DEGs between E+ and E− plants were much smaller compared to that between T400 and S279, showing generally higher transcript levels in E+ than E− plants, especially in the shoot (Figure 6c). The endophyte effect on root gene expression was very small in both T400 and S279 (Figure 6c).

Gene Ontology (GO) enrichment analysis was performed on drought‐regulated genes, which revealed that the following processes were induced under drought stress in all plant types: response to abiotic stresses (temperature, heat, high light, desiccation, salinity, cold, oxidative) and catabolism of organic acids, amino acids, cofactors, porphyrin‐containing compounds, and tetrapyrrole/chlorophyll. In contrast, genes associated with photosynthesis, biotic stress response (chitin), growth (response to nitrogen), receptor signaling pathways, phosphorylation and phosphate metabolism were substantially repressed under drought conditions (Table S2).

Next, GO enrichment analyses were performed on genes that were differentially expressed in T400 and S279, and between E+ and E−. When looking at the GO enrichment of genes that were differentially expressed in T400E− and S279E−, which presumably reflect intrinsic genetic differences between T400 and S279, five categories of genes were found to be enriched in the shoot, but none in the root (Table S3). Enriched genes involved in (programmed) cell death were generally more highly expressed in T400 shoots than in S279, under both well‐watered and stressed conditions. On the other hand, enriched genes related to response to chitin and nitrogen compound typically had lower expression levels in T400 shoots than in S279 under well‐watered conditions (Table S3). When comparing E+ and E− treatments, the presence of endophyte affected T400 and S279 in similar ways under drought stress, primarily by stimulating gene expression related to protein and nitrogen compound metabolism in the shoot (Table S3). However, under well‐watered conditions, similar endophyte effects on protein and nitrogen compound metabolism were observed only in T400 and not in S279. On the other hand, biotic stress responsive genes (chitin) and genes responding to nitrogen compounds (growth) were expressed at higher levels in E+ than E− roots in S279 under drought stress (Table S3).

When combining the genotype and endophyte effects and comparing between T400E+ and S279E+, we found that genes involved in degradation of organic acids and amino acids were enriched among the genes that had higher expression levels in drought‐stressed T400E+ than S279E+ in the shoot, but no significant category enrichment was identified in up‐regulated genes (T400E+/S279E+) in roots (Table S3). In contrast, strong enrichment was observed in the shoot down‐regulated genes (T400E+/S279E+), many in categories related to photosynthesis activity that responded to drought stress, i.e. light reaction, porphyrin‐containing compound biosynthesis/metabolism, tetrapyrrole biosynthesis/metabolism, photosynthetic electron transport, plastid organization, and chlorophyll biosynthesis (Table S3).

## 4 DISCUSSION

Plant drought adaptation and resistance include three main strategies: drought escape, drought avoidance, and drought tolerance (Aslam et al., 2015; Levitt, 2015). Drought escape refers to plants that alter their life cycle by either entering dormancy or flowering early when faced with drought stress (Kramer, 1980). Drought avoidance is related to a plant's ability to maintain high water potential under water limitation, mostly by reducing leaf transpiration and/or enhanced root growth (Levitt, 2015). In contrast, plant drought‐tolerance is primarily related to maintaining water uptake by accumulating osmolites under drought stress (Levitt, 2015). Earlier studies indicate that tall fescue uses all three strategies to survive drought stress. It is well known that Mediterranean tall fescue can enter summer dormancy in dry and hot environments, which is a typical mechanism of drought escape (Volaire & Norton, 2006). Under drought, tall fescue plants tend to develop deeper roots and larger root systems, an important mechanism for drought survival that was shown repeatedly to be associated with drought tolerance among different varieties (Carrow, 1996; Huang & Fry, 1998; Pirnajmedin et al., 2015). Past studies also showed that drought tolerant tall fescue cultivars contain higher protein and soluble carbohydrate content, and lower H_2_O_2_ content than sensitive ones (Rohollahi et al., 2018). For osmotic adjustment, multiple studies reported sharp increase of proline in tall fescue leaves under drought stress (Ebrahimiyan, Majidi, & Mirlohi, 2013; Pirnajmedin et al., 2017; Rohollahi et al., 2018; Sarmast et al., 2015). The role of other osmolytes such as sugar alcohols were much less studied (Bacon, 1993).

In the current study, we compared drought responses of two contrasting tall fescue genotypes and found that the drought tolerant genotype, T400, showed morphological and physiological characteristics related to drought avoidance and are typical for plants that are adapted to dry environments, e.g. small, narrow, but thick leaves, and relatively lower leaf conductance compared to the sensitive genotype, S279 (Figure1). This phenomenon has been reported broadly in grasses and other plant species, and these plants are generally called “water savers” (Kang et al., 2011; Maricle et al., 2007; Polania et al., 2016). Although leaf traits of E+ and E− plants were similar in both T400 and S279, endophyte symbiosis affected plant biomass differently in T400 and S279. Under both well‐watered (Figure 1) and drought (Figure S3) conditions, S279E+ plants had significantly higher root biomass but similar shoot biomass compared to S279E−. In T400, endophyte infection did not promote root growth significantly under either conditions (Figure 1; Figure S3). However, after severe drought stress and recovery, T400E+ had much larger shoot and root biomass than T400E−, revealing a delayed effect of endophyte during drought and recovery.

In earlier studies, endophyte symbiosis has been shown to promote plant growth and improve drought resistance (Feng et al., 2006; Khan et al., 2014). In tall fescue, endophyte presence was reported to increase shoot biomass, tiller numbers, and survival under field drought stress, while the benefit was not noticeable during wet years (West et al., 1993). In another study using three tall fescue genotypes and multiple endophyte species, significant genotype × endophyte interactions (*p* < .001) were observed for tiller density and shoot dry weight per area, indicating the promoting effect of endophyte on plant growth is association‐specific (Elbersen & West, 1996). Similar tall fescue cultivar × endophyte interaction was found in a separate study with elite cultivars infected with elite endophytes performing the best, and endophyte was more important in conferring resistance than difference between cultivars (Hume & Sewell, 2014). Therefore, the interaction between specific tall fescue and endophyte genotype appears to be important for the outcome. Here, we demonstrate that T400 and S279 responded to the same endophyte infection differently at the levels of phenology, physiology, molecular and biochemistry, and endophyte infection is crucial in enabling drought tolerance in T400, as discussed further below.

At molecular level, GO enrichment analysis revealed that genes related to photosynthesis were expressed at lower levels in T400E+ than in S279E+ (Table S3) under drought stress, consistent with a conservative strategy of T400E+ with respect to photosynthesis and linked transpiration. However, despite the drop in photosynthesis, reflected by the decline in biomass under drought stress (Figure 1; Figure S3), plants accumulated osmolytes especially proline, apparently via increased synthesis (Tables 1 and 2; Figure S1). Under both well‐watered and drought stressed conditions, T400E+ was much more active in protein biosynthesis and metabolism than T400E− (Table S3). Together, these observations may explain why T400E+ had the largest root and shoot biomass after severe drought stress and recovery (Figure 4). Our study confirms that the presence of endophyte has a positive effect on root growth and drought stress tolerance, as reported earlier in tall fescue (Arachevaleta et al., 1989; Bacon, 1993; West et al., 1993). In addition, T400 and S279 responded to endophyte differently in multiple levels (Figures 1, 3, and 4; Table 1), presumably due to plant genotype‐specific reactions to endophyte infection as reported earlier in tall fescue (Elbersen & West, 1996; Hume & Sewell, 2014).

As mentioned above, we observed a significant difference between plant genotypes in leaf osmotic pressure changes during drought, with T400 having a much larger leaf osmotic pressure increase under drought stress compared to S279 (Figure 3b). Higher leaf osmotic pressure indicates stronger osmotic adjustment and more osmolite accumulation, which is crucial for surviving drought stress and has been reported in tall fescue (West et al., 1990). Compared with drought‐adaptive phenotypic changes, e.g. smaller and thinker leaves, and lower stomatal density, osmotic adjustment is inducible and temporary. Therefore, it generally has less negative effect on growth and is more cost‐effective to plants (Johnson & Asay, 1993; McCree, 1986). Metabolite profiling confirmed greater accumulation of specific metabolites under severe drought stress in T400 than in S279 shoots (Table 1), especially organic acids. Consistent with this, GO enrichment analysis revealed genes involved in amino acid and organic acid catabolism amongst those with higher expression levels in T400 than S279 under drought stress (Table S3).

Among all metabolites detected, proline accumulated much more in T400 than in S279 under severe drought stress, in both roots and shoots, and both E+ and E− (Tables 1 and 2). In T400E+, proline content increased from 0.16 to 8.16 mg/g DW in the shoot, equivalent to a change in OP of 69.5 mmol/kg, explaining much of the leaf osmotic pressure increase under drought stress (Figure 3b). Transcript levels of one of the two proline biosynthetic enzymes, P5CS, mirrored those of proline content, consistent with P5CS being a rate‐limiting enzyme in proline biosynthesis (Delauney & Verma, 1993). Early studies demonstrated that over‐expression of P5CS in multiple plant species promotes proline biosynthesis and improves drought tolerance (Amini et al., 2015; Kavi Kishor et al., 1995; Vendruscolo et al., 2007; Yamchi et al., 2007). Similar association between proline accumulation, P5CS induction, and genotype drought sensitivity was reported in rice (Choudhary et al., 2005), *Brassica juncea* (Phutela et al., 2000), and wheat (Maghsoudi et al., 2018). However, proline accumulation was found not to be associated with genotype drought tolerance in Arabidopsis (Marín‐de la Rosa et al., 2019), alfalfa (Kang et al., 2011), and Tibetan hulless barley (Deng et al., 2013). Increased proline content does not necessarily associate with improved drought tolerance either (Pospisilova et al., 2011). Therefore, while proline is undoubtedly an important drought osmolite in plants, it may not be a universal marker for plant drought tolerance. In tall fescue, we observed contrasting patterns of proline accumulation associated with drought tolerance in the two genotypes, with more proline accumulated in the tolerant genotype. An earlier study in tall fescue obtained similar results with tolerant cultivar ‘Van Gogh’ accumulating 32% more leaf proline than the sensitive cultivar ‘AST7002’ under drought (Man et al., 2011). In the future, it would be interesting to expand this study to more genotypes and test the potential role of proline as a biochemical signature in screening for drought tolerance in tall fescue.

## 5 SUMMARY

In summary, gradual soil drought stress was applied to two tall fescue genotypes (T400 and S279) with contrasting drought tolerance, either with or without endophyte symbiosis. Physiological and biochemical analysis indicate that T400 (tolerant genotype) utilizes both drought escape and drought tolerance strategies to confer greater drought tolerance than S279 (sensitive genotype), for example, thicker and narrower leaves, lower transpiration, and more osmoticum especially proline accumulation under drought stress. Metabolite analysis with GC‐MS identified common and unique metabolites altered by drought stress in T400 and S279, with or without endophyte symbiosis. GO enrichment analysis of transcriptome changes revealed that the drought tolerant genotype, T400, repressed more genes related to photosynthesis and induced more genes related to organic acid and amino acid metabolism than the sensitive genotype. GO enrichment analysis also highlighted the role of endophyte in stimulating protein biosynthesis and metabolism in both genotypes.

## CONFLICT OF INTEREST

The authors have no conflict of interests to declare.

## Supporting information

Fig S1

Fig S2

Fig S3

Table S1‐3

Supplementary Material

## Data Availability

The data that support the findings of this study are openly available in the NCBI Sequence Read Archive at https://www.ncbi.nlm.nih.gov/sra, reference number PRJNA746971.

## References

[pei310066-bib-0001] Amini, S. , Ghobadi, C. , & Yamchi, A. (2015). Proline accumulation and osmotic stress: An overview of P5CS gene in plants. Journal of Plant Molecular Breeding, 3(2), 44–55. 10.22058/jpmb.2015.17022

[pei310066-bib-0002] An, Z. Q. , Siegel, M. R. , Hollin, W. , Tsai, H. F. , Schmidt, D. , & Schardl, C. L. (1993). Relationships among non‐*Acremonium* sp. fungal endophytes in five grass species. Applied and Environmental Microbiology, 59, 1540–1548. 10.1128/aem.59.5.1540-1548.1993 8517749 PMC182116

[pei310066-bib-0003] Arachevaleta, M. , Bacon, C. , Hoveland, C. , & Radcliffe, D. (1989). Effect of the tall fescue endophyte on plant response to environmental stress. Agronomy Journal, 81(1), 83–90. 10.2134/agronj1989.00021962008100010015x

[pei310066-bib-0004] Aslam, M. , Maqbool, M. A. , & Cengiz, R. (2015). Mechanisms of drought resistance. In M. Aslam , M. A. Maqbool , & R. Cengiz (Eds.), Drought stress in maize (*Zea mays* L.) (pp. 19–36). Springer.

[pei310066-bib-0005] Assuero, S. G. , Matthew, C. , Kemp, P. , Barker, D. J. , & Mazzanti, T. L. A. (2002). Effects of water deficit on Mediterranean and temperate cultivars of tall fescue. Australian Journal of Agricultural Research, 53(1), 29–40. 10.1071/AR01023

[pei310066-bib-0006] Bacon, C. W. (1993). Abiotic stress tolerances (moisture, nutrients) and photosynthesis in endophyte‐infected tall fescue. Agriculture, Ecosystems & Environment, 44(1–4), 123–141. 10.1016/0167-8809(93)90042-N

[pei310066-bib-0007] Bates, L. S. , Waldren, R. P. , & Teare, I. (1973). Rapid determination of free proline for water‐stress studies. Plant and Soil, 39(1), 205–207. 10.1007/BF00018060

[pei310066-bib-0008] Benny, J. , Pisciotta, A. , Caruso, T. , & Martinelli, F. (2019). Identification of key genes and its chromosome regions linked to drought responses in leaves across different crops through meta‐analysis of RNA‐Seq data. BMC Plant Biology, 19(1), 194. 10.1186/s12870-019-1794-y 31077147 PMC6511156

[pei310066-bib-0009] Bouton, J. , Gates, R. , Belesky, D. , & Owsley, M. (1993). Yield and persistence of tall fescue in the southeastern coastal plain after removal of its endophyte. Agronomy Journal, 85(1), 52–55. 10.2134/agronj1993.00021962008500010011x

[pei310066-bib-0010] Carrow, R. (1996). Drought avoidance characteristics of diverse tall fescue cultivars. Crop Science, 36(2), 371–377. 10.2135/cropsci1996.0011183X003600020026x

[pei310066-bib-0011] Chen, Z. , Wang, Z. , Yang, Y. , Li, M. , & Xu, B. (2018). Abscisic acid and brassinolide combined application synergistically enhances drought tolerance and photosynthesis of tall fescue under water stress. Scientia Horticulturae, 228, 1–9. 10.1016/j.scienta.2017.10.004

[pei310066-bib-0012] Choudhary, N. , Sairam, R. & Tyagi, A. (2005). Expression of Δ¹‐pyrroline‐5‐carboxylate synthetase gene during drought in rice (Oryza sativa L.). Indian Journal of Biochemistry and Biophysics, 42(6), 366‐370. 16955737

[pei310066-bib-0013] de Bruijn, F. J. (2020). The model legume *Medicago* *truncatula*, 2 volume set. John Wiley & Sons.

[pei310066-bib-0014] Delauney, A. J. , & Verma, D. P. S. (1993). Proline biosynthesis and osmoregulation in plants. The Plant Journal, 4(2), 215–223. 10.1046/j.1365-313X.1993.04020215.x

[pei310066-bib-0015] Deng, G. , Liang, J. , Xu, D. , Long, H. , Pan, Z. , & Yu, M. (2013). The relationship between proline content, the expression level of P5CS (Δ 1‐pyrroline‐5‐carboxylate synthetase), and drought tolerance in Tibetan hulless barley (*Hordeum* *vulgare* var. nudum). Russian Journal of Plant Physiology, 60(5), 693–700. 10.1134/S1021443713050038

[pei310066-bib-0016] Dinkins, R. D. , Nagabhyru, P. , Young, C. A. , West, C. P. , & Schardl, C. L. (2019). Transcriptome analysis and differential expression in tall fescue harboring different endophyte strains in response to water deficit. The Plant Genome, 12(2), 180071. 10.3835/plantgenome2018.09.0071 PMC12810098 31290925

[pei310066-bib-0017] Ebrahimiyan, M. , Majidi, M. , & Mirlohi, A. (2013). Genotypic variation and selection of traits related to forage yield in tall fescue under irrigated and drought stress environments. Grass and Forage Science, 68(1), 59–71. 10.1111/j.1365-2494.2012.00869.x

[pei310066-bib-0018] Ebrahimiyan, M. , Majidi, M. M. , Mirlohi, A. , & Noroozi, A. (2013). Physiological traits related to drought tolerance in tall fescue. Euphytica, 190(3), 401–414. 10.1007/s10681-012-0808-8

[pei310066-bib-0019] Egea, I. , Albaladejo, I. , Meco, V. , Morales, B. , Sevilla, A. , Bolarin, M. C. , & Flores, F. B. (2018). The drought‐tolerant *Solanum* *pennellii* regulates leaf water loss and induces genes involved in amino acid and ethylene/jasmonate metabolism under dehydration. Scientific Reports, 8(1), 1–14. 10.1038/s41598-018-21187-2 29434236 PMC5809557

[pei310066-bib-0020] Elbersen, H. , & West, C. (1996). Growth and water relations of field‐grown tall fescue as influenced by drought and endophyte. Grass and Forage Science, 51(4), 333–342. 10.1111/j.1365-2494.1996.tb02068.x

[pei310066-bib-0021] Elmi, A. , West, C. , Robbins, R. , & Kirkpatrick, T. (2000). Endophyte effects on reproduction of a root‐knot nematode (*Meloidogyne* *marylandi*) and osmotic adjustment in tall fescue. Grass and Forage Science, 55(2), 166–172. 10.1046/j.1365-2494.2000.00210.x

[pei310066-bib-0022] Fang, Y. , & Xiong, L. (2015). General mechanisms of drought response and their application in drought resistance improvement in plants. Cellular and Molecular Life Sciences, 72(4), 673–689. 10.1007/s00018-014-1767-0 25336153 PMC11113132

[pei310066-bib-0023] Farooq, M. , Hussain, M. , Wahid, A. , & Siddique, K. (2012). Drought stress in plants: An overview. In R. Aroca (Ed.), Plant responses to drought stress (pp. 1–33). Springer.

[pei310066-bib-0024] Feng, Y. , Shen, D. , & Song, W. (2006). Rice endophyte *Pantoea* *agglomerans* YS19 promotes host plant growth and affects allocations of host photosynthates. Journal of Applied Microbiology, 100(5), 938–945. 10.1111/j.1365-2672.2006.02843.x 16629994

[pei310066-bib-0025] Fornes, O. , Castro‐Mondragon, J. A. , Khan, A. , Van der Lee, R. , Zhang, X. , Richmond, P. A. , & Baranašić, D. (2020). JASPAR 2020: Update of the open‐access database of transcription factor binding profiles. Nucleic Acids Research, 48(D1), D87–D92. 10.1093/nar/gkz1001 31701148 PMC7145627

[pei310066-bib-0026] Fracasso, A. , Trindade, L. M. , & Amaducci, S. (2016). Drought stress tolerance strategies revealed by RNA‐Seq in two sorghum genotypes with contrasting WUE. BMC Plant Biology, 16(1), 1–18. 10.1186/s12870-016-0800-x 27208977 PMC4875703

[pei310066-bib-0027] Ge, S. X. , Jung, D. , & Yao, R. (2020). ShinyGO: A graphical gene‐set enrichment tool for animals and plants. Bioinformatics, 36(8), 2628–2629. 10.1093/bioinformatics/btz931 31882993 PMC7178415

[pei310066-bib-0028] Hamid, H. , Khaedr, A. , Mohammad, A. , Amal, A. , Quick, P. , & Abogadallah, M. (2003). Proline induces the expression of salt–stress–responsive proteins and may improve the adoption of *Pancratium* *maritimum* L. to salt stress. Journal of Experimental Botany, 54, 2553–2562. 10.1093/jxb/erg277 14512386

[pei310066-bib-0029] Hirayama, T. , & Shinozaki, K. (2010). Research on plant abiotic stress responses in the post‐genome era: Past, present and future. The Plant Journal, 61(6), 1041–1052. 10.1111/j.1365-313X.2010.04124.x 20409277

[pei310066-bib-0030] Hopkins, A. A. , Young, C. A. , Butler, T. J. , & Bouton, J. H. (2011). Registration of ‘Texoma’MaxQ II tall fescue. Journal of Plant Registrations, 5(1), 14–18. 10.3198/jpr2010.02.0082crc

[pei310066-bib-0031] Huang, B. , & Fry, J. D. (1998). Root anatomical, physiological, and morphological responses to drought stress for tall fescue cultivars. Crop Science, 38(4), 1017–1022. 10.2135/cropsci1998.0011183X003800040022x

[pei310066-bib-0032] Huang, B. , & Gao, H. (2000). Root physiological characteristics associated with drought resistance in tall fescue cultivars. Crop Science, 40(1), 196–203. 10.2135/cropsci2000.401196x

[pei310066-bib-0033] Hume, D. , & Sewell, J. (2014). Agronomic advantages conferred by endophyte infection of perennial ryegrass (*Lolium* *perenne* L.) and tall fescue (*Festuca* *arundinacea* Schreb.) in Australia. Crop and Pasture Science, 65(8), 747–757. 10.1071/CP13383

[pei310066-bib-0034] Johnson, D. A. , & Asay, K. H. (1993). Selection for improved drought response in cool‐season grasses. Rangeland Ecology & Management/Journal of Range Management Archives, 46(3), 194–202. 10.2307/4002606

[pei310066-bib-0035] Kang, Y. , Han, Y. , Torres‐Jerez, I. , Wang, M. , Tang, Y. , Monteros, M. J. , & Udvardi, M. K. (2011). System responses to long‐term drought and re‐watering of two contrasting alfalfa varieties. The Plant Journal, 68(5), 871–889. 10.1111/j.1365-313X.2011.04738.x 21838776

[pei310066-bib-0036] Kavi Kishor, K. P. , Hong, Z. , Miao, G.‐H. , Hu, C. , & Verma, D. P. S. (1995). Overexpression of delta 1‐pyrroline‐5‐carboxylate synthetase increases proline production and confers osmotolerance in transgenic plants. Plant Physiology, 108, 1387–1394. 10.1104/pp.108.4.1387 12228549 PMC157516

[pei310066-bib-0037] Khan, A. L. , Waqas, M. , Kang, S.‐M. , Al‐Harrasi, A. , Hussain, J. , Al‐Rawahi, A. , & Jung, H.‐Y. (2014). Bacterial endophyte Sphingomonas sp. LK11 produces gibberellins and IAA and promotes tomato plant growth. Journal of Microbiology, 52(8), 689–695. 10.1007/s12275-014-4002-7 24994010

[pei310066-bib-0038] Kramer, P. J. (1980). Drought stress and the origin of adaptations. In N. C. Turner , & P. J. Kramer (Eds.), Adaptation of plants to water and high temperature stress (pp. 7–30). John Wiley & Sons.

[pei310066-bib-0039] Levitt, J. (2015). Water, radiation, salt, and other stresses (Vol. 2). Elsevier.

[pei310066-bib-0040] Maghsoudi, K. , Emam, Y. , Niazi, A. , Pessarakli, M. , & Arvin, M. J. (2018). P5CS expression level and proline accumulation in the sensitive and tolerant wheat cultivars under control and drought stress conditions in the presence/absence of silicon and salicylic acid. Journal of Plant Interactions, 13(1), 461–471. 10.1080/17429145.2018.1506516

[pei310066-bib-0041] Malinowski, D. P. , & Belesky, D. P. (2000). Adaptations of endophyte‐infected cool‐season grasses to environmental stresses: Mechanisms of drought and mineral stress tolerance. Crop Science, 40(4), 923–940. 10.2135/cropsci2000.404923x

[pei310066-bib-0042] Man, D. , Bao, Y.‐X. , Han, L.‐B. , & Zhang, X. (2011). Drought tolerance associated with proline and hormone metabolism in two tall fescue cultivars. HortScience, 46(7), 1027–1032. 10.21273/HORTSCI.46.7.1027

[pei310066-bib-0043] Maricle, B. R. , Cobos, D. R. , & Campbell, C. S. (2007). Biophysical and morphological leaf adaptations to drought and salinity in salt marsh grasses. Environmental and Experimental Botany, 60(3), 458–467. 10.1016/j.envexpbot.2007.01.001

[pei310066-bib-0044] Marín‐de la Rosa, N. , Lin, C. W. , Kang, Y. J. , Dhondt, S. , Gonzalez, N. , Inzé, D. , & Falter‐Braun, P. (2019). Drought resistance is mediated by divergent strategies in closely related Brassicaceae. New Phytologist, 223(2), 783–797. 10.1111/nph.15841 30955214 PMC6771540

[pei310066-bib-0045] McCree, K. (1986). Whole‐plant carbon balance during osmotic adjustment to drought and salinity stress. Functional Plant Biology, 13(1), 33–43. 10.1071/PP9860033

[pei310066-bib-0046] Meena, Y. K. , & Kaur, N. (2019). Towards an understanding of physiological and biochemical mechanisms of drought tolerance in plant. Annual Research & Review in Biology, 1–13. 10.9734/arrb/2019/v31i230042

[pei310066-bib-0047] Monneveux, P. , & Belhassen, E. (1996). The diversity of drought adaptation in the wide. In E. Belhassen (Ed.), Drought tolerance in higher plants: Genetical, physiological and molecular biological analysis (pp. 7–14). Springer.

[pei310066-bib-0048] Nagabhyru, P. , Dinkins, R. D. , Wood, C. L. , Bacon, C. W. , & Schardl, C. L. (2013). Tall fescue endophyte effects on tolerance to water‐deficit stress. BMC Plant Biology, 13, 127. 10.1186/1471-2229-13-127 24015904 PMC3848598

[pei310066-bib-0049] Panaccione, D. G. , Cipoletti, J. R. , Sedlock, A. B. , Blemings, K. P. , Schardl, C. L. , Machado, C. , & Seidel, G. E. (2006). Effects of ergot alkaloids on food preference and satiety in rabbits, as assessed with gene‐knockout endophytes in perennial ryegrass (*Lolium* *perenne*). Journal of Agricultural and Food Chemistry, 54(13), 4582–4587. 10.1021/jf060626u 16787001

[pei310066-bib-0050] Pedersen, J. F. , Lacefield, G. & Ball, D. (1990). A review of the agronomic characteristics of endophyte‐free and endophyte‐infected tall fescue. Applied Agricultural Research, 5(3),188‐194.

[pei310066-bib-0051] Phutela, A. , Jain, V. , Dhawan, K. , & Nainawatee, H. (2000). Proline metabolism under water stress in the leaves and roots of *Brassica* *juncea* cultivars differing in drought tolerance. Journal of Plant Biochemistry and Biotechnology, 9(1), 35–39. 10.1007/BF03263081

[pei310066-bib-0052] Pirnajmedin, F. , Majidi, M. M. , & Gheysari, M. (2015). Root and physiological characteristics associated with drought tolerance in Iranian tall fescue. Euphytica, 202(1), 141–155. 10.1007/s10681-014-1239-5

[pei310066-bib-0053] Pirnajmedin, F. , Majidi, M. M. , Saeidi, G. , Gheysari, M. , Nourbakhsh, V. , & Radan, Z. (2017). Genetic analysis of root and physiological traits of tall fescue in association with drought stress conditions. Euphytica, 213(7), 1–16. 10.1007/s10681-017-1920-6

[pei310066-bib-0054] Polania, J. A. , Poschenrieder, C. , Beebe, S. , & Rao, I. M. (2016). Effective use of water and increased dry matter partitioned to grain contribute to yield of common bean improved for drought resistance. Frontiers in Plant Science, 7. 10.3389/fpls.2016.00660 PMC4864351 27242861

[pei310066-bib-0055] Pospisilova, J. , Haisel, D. , & Vankova, R. (2011). Responses of transgenic tobacco plants with increased proline content to drought and/or heat stress. American Journal of Plant Sciences, 2(03), 318–324. 10.4236/ajps.2011.23036

[pei310066-bib-0056] Rohollahi, I. , Khoshkholghsima, N. , Nagano, H. , Hoshino, Y. , & Yamada, T. (2018). Respiratory burst oxidase‐D expression and biochemical responses in *Festuca* *arundinacea* under drought stress. Crop Science, 58(1), 435–442. 10.2135/cropsci2017.07.0416

[pei310066-bib-0057] Saha, M. C. , Talukder, S. , Azhaguvel, P. , Mukhergee, S. , & Chekhovskiy, K. (2015). Deciphering drought tolerance in tall fescue [*Lolium* *arundinaceum* (Schreb.) Darbysh.]. In A. Hopkins , Z. Wang , R. Mian , M. Sledge , & R. E. Barker (Eds.), Molecular breeding of forage and turf (pp. 1–7). Springer.

[pei310066-bib-0058] Sarmast, M. , Salehi, H. , & Niazi, A. (2015). Biochemical differences underlie varying drought tolerance in four *Festuca* *arundinacea* Schreb. genotypes subjected to short water scarcity. Acta Physiologiae Plantarum, 37(9), 1–13.

[pei310066-bib-0059] Sheffer, K. , Dunn, J. , & Minner, D. (1987). Summer drought response and rooting depth of three cool‐season turfgrasses. HortScience, 22(2), 296–297.

[pei310066-bib-0060] Sleper, D. , & West, C. (1996). Tall fescue. In L. E. Moser , D. R. Buxton , & M. D. Casler (Eds.), Cool‐season forage grasses (Vol. 34, pp. 471–502). American Society of Agronomy, Crop Science Society of America, Soil Science Society of America.

[pei310066-bib-0061] Sun, J. , Meyer, W. , Cross, J. , & Huang, B. (2013). Growth and physiological traits of canopy and root systems associated with drought resistance in tall fescue. Crop Science, 53(2), 575–584. 10.2135/cropsci2012.05.0292

[pei310066-bib-0062] Sun, L. , Dong, S. , Ge, Y. , Fonseca, J. P. , Robinson, Z. T. , Mysore, K. S. , & Mehta, P. (2019). DiVenn: An interactive and integrated web‐based visualization tool for comparing gene lists. Frontiers in Genetics, 10, 421. 10.3389/fgene.2019.00421 31130993 PMC6509638

[pei310066-bib-0063] Takach, J. E. , Mittal, S. , Swoboda, G. A. , Bright, S. K. , Trammell, M. A. , Hopkins, A. A. , & Young, C. A. (2012). Genotypic and chemotypic diversity of Neotyphodium endophytes in tall fescue from Greece. Applied and Environmental Microbiology, 78, 5501–5510. 10.1128/AEM.01084-12 22660705 PMC3406137

[pei310066-bib-0064] Talukder, S. , Azhaguvel, P. , Mukherjee, S. , Young, C. , Tang, Y. , Krom, N. , & Saha, M. (2015). De novo assembly and characterization of tall fescue transcriptome under water stress. The Plant Genome, 8(2). 10.3835/plantgenome2014.09.0050 33228317

[pei310066-bib-0065] Tian, T. , Liu, Y. , Yan, H. , You, Q. , Yi, X. , Du, Z. , Xu, W. , & Su, Z. (2017). agriGO v2.0: A GO analysis toolkit for the agricultural community, 2017 update. Nucleic Acids Research, 45(W1), W122–W129. 10.1093/nar/gkx382 28472432 PMC5793732

[pei310066-bib-0066] Uno, Y. , Furihata, T. , Abe, H. , Yoshida, R. , Shinozaki, K. , & Yamaguchi‐Shinozaki, K. (2000). Arabidopsis basic leucine zipper transcription factors involved in an abscisic acid‐dependent signal transduction pathway under drought and high‐salinity conditions. Proceedings of the National Academy of Sciences of the United States of America, 97(21), 11632–11637. 10.1073/pnas.190309197 11005831 PMC17252

[pei310066-bib-0067] Vendruscolo, E. C. G. , Schuster, I. , Pileggi, M. , Scapim, C. A. , Molinari, H. B. C. , Marur, C. J. , & Vieira, L. G. E. (2007). Stress‐induced synthesis of proline confers tolerance to water deficit in transgenic wheat. Journal of Plant Physiology, 164(10), 1367–1376. 10.1016/j.jplph.2007.05.001 17604875

[pei310066-bib-0068] Volaire, F. , & Norton, M. (2006). Summer dormancy in perennial temperate grasses. Annals of Botany, 98(5), 927–933. 10.1093/aob/mcl195 17028299 PMC2803600

[pei310066-bib-0069] Wang, P. , Yang, C. , Chen, H. , Song, C. , Zhang, X. , & Wang, D. (2017). Transcriptomic basis for drought‐resistance in *Brassica* *napus* L. Scientific Reports, 7(1), 1–20. 10.1038/srep40532 28091614 PMC5238399

[pei310066-bib-0070] West, C. , Izekor, E. , Turner, K. , & Elmi, A. (1993). Endophyte effects on growth and persistence of tall fescue along a water‐supply gradient. Agronomy Journal, 85(2), 264–270. 10.2134/agronj1993.00021962008500020019x

[pei310066-bib-0071] West, C. , Oosterhuis, D. , & Wullschleger, S. (1990). Osmotic adjustment in tissues of tall fescue in response to water deficit. Environmental and Experimental Botany, 30(2), 149–156. 10.1016/0098-8472(90)90059-D

[pei310066-bib-0072] Yamchi, A. , Rastgar Jazii, F. , Mousavi, A. , Karkhane, A. A. , & Renu, (2007). Proline accumulation in transgenic tobacco as a result of expression of Arabidopsis Δ 1‐pyrroline‐5‐carboxylate synthetase (P5CS) during osmotic stress. Journal of Plant Biochemistry and Biotechnology, 16(1), 9–15. 10.1007/BF03321922

