# Supplementary figures and images for: Dissection of physiological, transcriptional, and metabolic traits in two tall fescue genotypes with contrasting drought tolerance
Source: Plant Environ Interact. 2021 Nov 22;2(6):277–89. doi: 10.1002/pei3.10066 (PMC10168078; doi:10.1002/pei3.10066)

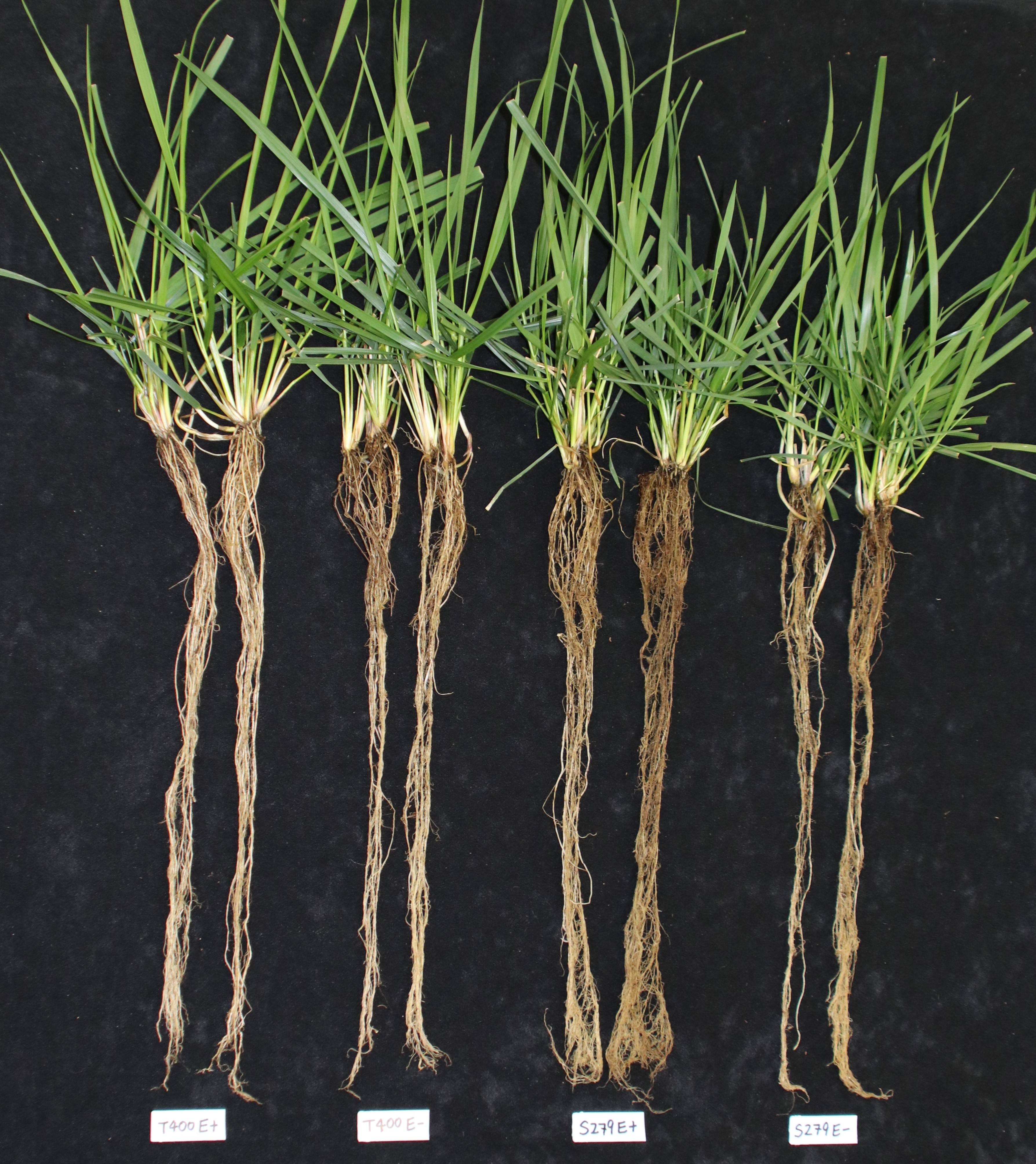

Supplement: Supplementary file 1 — Fig S1 [file PEI3-2-277-s002.jpg]

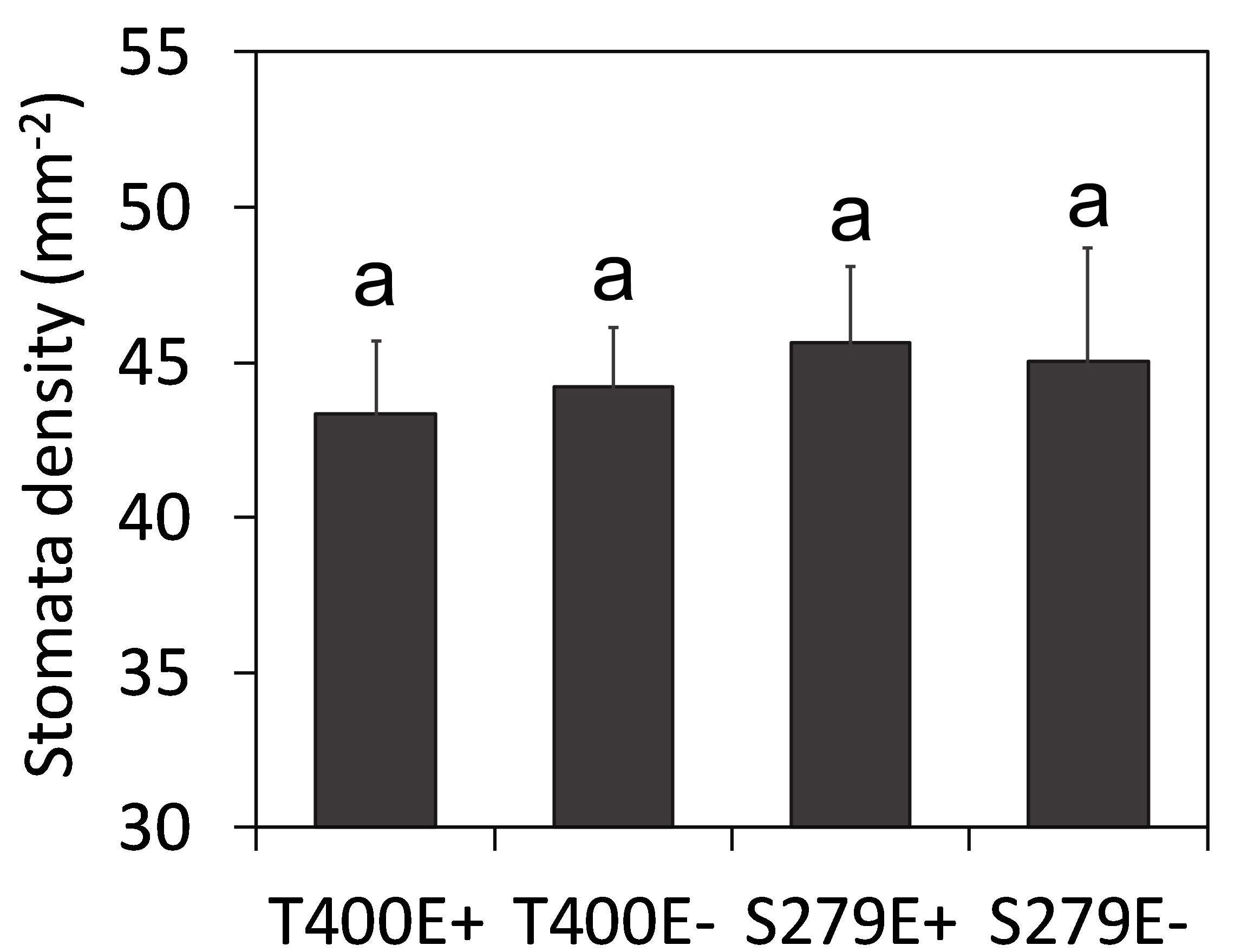

Supplement: Supplementary file 2 — Fig S2 [file PEI3-2-277-s003.jpg]

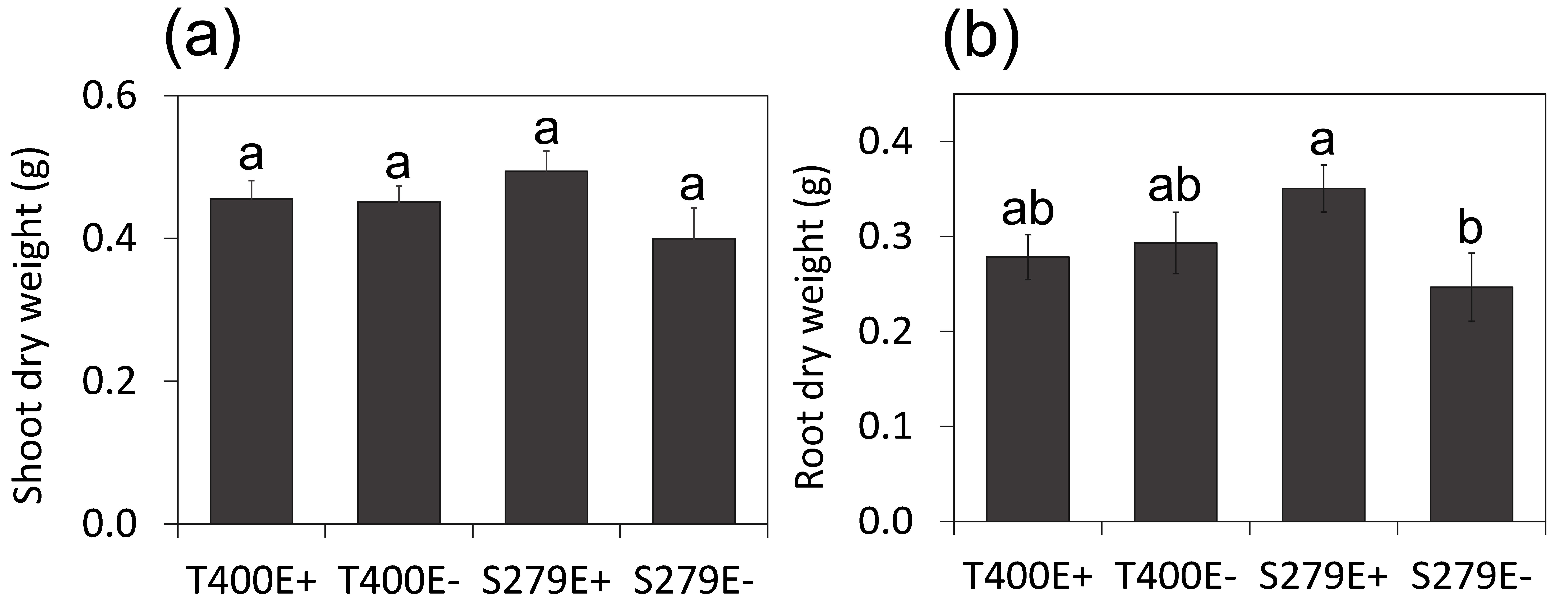

Supplement: Supplementary file 3 — Fig S3 [file PEI3-2-277-s001.jpg]
