# Supplementary material for: Dissection of physiological, transcriptional, and metabolic traits in two tall fescue genotypes with contrasting drought tolerance
Source: Plant Environ Interact. 2021 Nov 22;2(6):277–89. doi: 10.1002/pei3.10066 (PMC10168078; doi:10.1002/pei3.10066)
Supplement: Supplementary file 5 — Supplementary Material [file PEI3-2-277-s004.docx]

**FIGURE S1.** Well-watered T400E+, T400E-, S279E+, and S279E- plants, from the left to the right.

**FIGURE S2.** Stomata density on the leaf abaxial side of well-watered tall fescue plants. Same letters indicate no significant difference at p < 0.05 (Duncan's Test), n=5, error bars are standard errors.

**FIGURE S3.** Shoot (a) and root (b) biomass of severely drought-stressed plants (DrtC). Different letters indicate significant difference at p < 0.05 (Duncan's Test), n=5, error bars are standard errors.

**TABLE S1.** Transcript (FPKM) changes, annotations, and sequences of major proline biosynthesis and degradation genes under drought stress.

**TABLE S2.** GO enrichment of differentially expressed genes (DEGs) between drought-stressed and well-watered plants with FCs > 2 and p_adj_ < 0.05.

**TABLE S3**. GO enrichment of differentially expressed genes (DEGs) between T400 and S279 and between endophytic (E+) and non-endophytic (E-) plants with FCs > 2 and p_adj_ < 0.05.
